# Tauopathy Differentially Affects Cell Adhesion Molecules in Mouse Brain: Early Down-Regulation of Nectin-3 in Stratum Lacunosum Moleculare

**DOI:** 10.1371/journal.pone.0063589

**Published:** 2013-05-21

**Authors:** Hervé Maurin, Claire Marie Seymour, Benoit Lechat, Peter Borghgraef, Herman Devijver, Tomasz Jaworski, Mathias V. Schmidt, Sebastian Kuegler, Fred Van Leuven

**Affiliations:** 1 Experimental Genetics Group - LEGTEGG, Dept Human Genetics, KULeuven, Leuven, Belgium; 2 Max Planck Institute of Psychiatry, Munich, Germany; 3 Molecular Physiology of the Brain (CMPB), Göttingen, Germany; Universidad de Sevilla, Spain

## Abstract

Cell adhesion molecules are important structural substrates, required for synaptic plasticity and synaptogenesis. CAMs differ widely in their expression throughout different brain regions and their specific structural and functional roles in the brain remain to be elucidated. Here, we investigated selected cell adhesion molecules for alterations in expression levels and neuronal localization in validated mouse models for Alzheimer's disease that mimic the age-related progression of amyloid accumulation and tauopathy. Among the cell adhesion molecules analyzed, Nectin-3 expression was affected most and specifically in all mouse models with tauopathy. In particular was Nectin-3 depleted from the specific region of the hippocampus, known as the stratum lacunosum and moleculare, in mice that express wild-type or mutant human protein Tau, either chronically or sub-acutely. Tauopathy progresses from the entorhinal cortex to the hippocampus by unknown mechanisms that could involve transport by the myelinated axons of the temporoammonic and perforant pathways. The decreased expression of Nectin-3 in the stratum lacunosum moleculare is an early marker of impaired transport, and eventual synaptic problems, caused by beginning tauopathy.

## Introduction

Alzheimer's Disease (AD) is the most prevalent form of dementia and its incidence and prevalence is increasing in our ageing populations [1]. Cognitive decline in AD was originally proposed to result from extracellular amyloid plaques and intraneuronal tauopathy, the two major pathological hallmarks in post-mortem AD-brain. More recently, defects in the structure and function of synapses were proposed as the underlying cause of the progressive cognitive decline associated with normal ageing and with dementia. Nevertheless, individuals with mild cognitive impairment (MCI) do not necessarily convert to AD, despite the reduced numbers of synapses in their hippocampal CA1 region [2]. Factors that cause the synaptic defects in MCI and the subsequent conversion to AD remain largely unknown. Accumulation of amyloid peptides occurs in MCI, and is even observed in normocognitive individuals. This is an important event in relation to the triggering of tauopathy, the co-morbid pathology always associated with amyloid accumulation in AD. The tauopathy component in AD is known to comprise aberrantly increased tau phosphorylation, which involves various kinases [3]–[5]. In particular, the role of activated glycogen synthase kinase-3 (GSK3) needs to be further elucidated also because of its important functions in normal synaptic transmission [6]–[9].

Learning and memory rely on the establishing and the strengthening of synapses and neural circuits, which need to be both stable and plastic [10]. Synaptic plasticity and spinogenesis depend on modifications of membrane adhesion properties, regulated by a wide variety of cell-adhesion molecules (CAMs). Synapses comprise two types of junctions and both are populated by different CAMs: (i) synaptic junctions with asymmetric profiles across the synaptic clefts, wherein CAMs maintain the structural integrity of the synapse and function in cell-cell signaling, and (ii) puncta adherentia junctions (PAJ) that are mostly symmetrically structured [11] with CAMs playing mechanical roles [12]–[15]. Problems with normal CAM functioning are suspected to contribute to synaptic dysfunction, which may eventually lead to neurodegeneration. Neurons within the entorhinal cortex (ERC) and Cornus Ammonis (CA) regions (Fig. S1) are the most vulnerable in AD, most likely due to these neurons harboring the causes of disease initiation and spreading [16]–[20]. Two major myelinated axonal tracks connect the ERC to the hippocampus: (i) the perforant pathway (PP) projecting from ERC layer II to dentate gyrus, and (ii) the temporoammonic pathway (TA) linking ERC layer III to CA1 by synapsing on apical dendrites within the stratum lacunosum moleculare (SLM) [21], [22]. The precise anatomical connections and the roles played by different CAMs in these pathways, in normal brain and in cognitive disorders such as AD, remain to be elucidated.

Here, we investigated a selection of CAMs for alterations in their expression and localization throughout the age-related progression of amyloid and tauopathy in different mouse models. These mouse models have been validated for specified aspects of AD [23], [24]. Among the CAMs analyzed, Nectin-3 expression was the most noticeably affected in the AD mouse models for tauopathy. Nectin-3 is a CAM predominantly expressed in PAJ and we observed it to be lacking in the SLM of mice expressing human protein Tau, either chronically by transgenesis or sub-acutely by intracerebral injection of adeno-associated virus (AAV)-vectors [5], [25]–[27].

## Materials and Methods

### Transgenic mice and AAV mouse models

All animal experiments were performed by certified researchers conforming to regional, national and European regulations concerning animal welfare and animal experimentation, authorized and supervised by the university animal welfare commission (Ethische Commissie Dierenwelzijn, KULeuven). We formally declare that we comply to the European FP7-Decision 1982/2006/EC, Article 611, i.e. all research activities is carried out in compliance with fundamental ethical principles and all experiments are approved and overlooked by the respective Animal Welfare Commissions.

The full details on the characterization and validation of the mouse models used in this study are available in the associated references. All the transgenic mice were generated in or back-crossed to the FvB genetic background. Four major genotypes were analyzed in depth in this study and were denoted according to their transgene(s): (i) Tau.P301L: mice expressing human tau protein containing the P301L mutation [25], (ii) APP.V717I: mice expressing amyloid precursor protein(APP) with the V717I mutation [28], [29], (iii) bigenic biAT: mice which are a cross of the APP.V717I x Tau.P301L mice [26], [30], and (iv) bigenic biGT: mice which are a cross of the Tau.P301L x GSK3β[S9A] mice [26]. For some experiments, these strains were compared to two other strains, mice expressing human Tau.4R [31] or mice expressing GSK3β[S9A], constitutively active GSK3β and denoted TG3 [32], [33]. Mice deficient in protein tau (Jackson labs, Bar Harbor, ME) were used as a negative control in some experiments [34].

We also analyzed FvB wild-type mice (age 4–7 months) injected unilaterally in their hippocampus at coordinates posterior 1.94 mm, lateral 1.4 mm, ventral 2.2 mm relative to bregma, with adeno-associated virus vectors (AAV) to express one of the following human proteins: wild-type Tau.4R, mutant Tau.P301L, C-truncated Tau (Tau.255) lacking the microtubule-binding domains, wild-type APP, and the triple mutant APP-SLA (Swedish-London-Austrian mutation) [5], [27]. Injection of AAV-Tau.4R in the entorhinal cortex was at coordinates posterior 4.72 mm, lateral 3.25 mm and 3.5 mm relative to bregma.

### Hippocampal synaptosomes

Hippocampi were rapidly dissected from mouse brains and homogenized to prepare synaptosomes [35]. Crude synaptosomal pellets were resuspended in 10 volumes of 50 mM HEPES (pH 7.4) containing 2 mM EDTA and proteinase and phosphatase inhibitors. Total homogenates and synaptosomal preparations were analyzed by western blotting, after proteins were denatured, reduced and separated on 10% Tris-Glycine SDS-PAGE gels (Anamed, Germany). After protein transfer, nitrocellulose membranes were blocked with skimmed milk (5% in TBS) and probed for: Nectin-3 (rabbit anti Nectin-3, AbCam 63931), Nectin-1 (rabbit anti Nectin-1, generous gift from H.J. Federoff, Georgetown University; [36]), NCAM (goat anti NCAM, AbCys AbC0026), and SynCAM (rabbit anti SynCAM, Sigma S4945) by overnight incubation with the primary antibodies at 4°C. After rinsing, membranes were incubated with suitable peroxidase-labeled secondary antibodies for 1 hr at room temperature. Development of immune reactions (ECL prime; GE Healthcare), image acquisition and analysis were performed as previously described (ImageQuant software v7.0; LAS 4000 imager, GE Healthcare) [35], [37].

### In situ hybridization

Mouse brains were quickly removed, frozen in isopentane cooled on dry ice, and stored at −80°C until analysis. In situ hybridization was performed as described [38], [39]. In brief, coronal brain sections (20 µm) were thaw-mounted on superfrost slides, dried and kept at −80°C. The following primers were used to generate antisense RNA hybridization probes (485 basepairs) recognizing shared sequences of Nectin-3 variants: AGCCGTTACATTCCCACTTG (forward primer) and ATTGTCCATCCAACCTGCTC (reverse primer). The riboprobe for Nectin-1(406 basepairs) was generated using the following primers: AGTCGGGTTGTAGATGGCCA (forward primer) and GTCATCAGCCGTTACCGTTT (reverse primer). After radiolabeling, hybridization of slides were apposed to film (Biomax MR, Eastman Kodak) and autoradiographs were digitized for determination of relative expression by dedicated software (Scion Image analysis).

### Immunohistochemistry (IHC)

Mice were anesthetized (Nembutal; 100 mg/kg, i.p.) before transcardiac perfusion with ice-cold saline (4 ml/min, for 2 min). Brains were quickly removed and fixed overnight in 4% paraformaldehyde in PBS at 4°C, and stored in PBS containing 0.1% sodium azide at 4°C until sectioning by vibratome (40 µm). Immunohistochemistry was performed as previously described [25]–[27], [35]. After rinsing in PBS, sections were pretreated for 15 min with 1.5% H_2_O_2_ in 50% methanol/PBS to eliminate endogenous brain peroxidase activity. Subsequent blocking of nonspecific binding sites by incubation in 10% fetal calf serum, 0.1% Triton X-100 in PBS (blocking solution), was followed by incubation at 4°C overnight with primary antibodies: AT180 (mouse anti-pT231, Thermo Scientific MN1040), Nectin-3 (rabbit anti Nectin-3, AbCam 63931), Nectin-1(generous gift from H.J. Federoff, Georgetown University; [36]), NCAM (goat anti NCAM, AbCys AbC0026), PSA-NCAM (mouse anti PSA-NCAM, AbCys AbC0019) or SynCAM (rabbit anti-SynCAM, Sigma S4945). After rinsing (0.1% TritonX-100 in PBS), sections were incubated for 1 h with the appropriate secondary antibodies diluted 1∶500 in blocking buffer. When indicated, sections were incubated for 30 min with avidin-biotin complex (Vectastain ABC Elite, Vector labs). Sections were rinsed in PBS and incubated for 5 min in 50 mM Tris-HCl (pH 7.6), before staining with 3,3′-diaminobenzidine (0.5 mg/ml), 0.3% H_2_O_2_ in 50 mM Tris-HCl (pH 7.6). Hematoxylin was used for counterstaining, prior to dehydration by passage through a graded series of ethanol-water mixtures. After incubation in 100% xylol, the dehydrated sections were mounted on microscope glass-slides (DePeX) [35]. For confocal microscopy, following overnight incubation with HT7 (mouse anti HT7, Thermo scientific MN1000) and Nectin-3 (rabbit anti Nectin-3, AbCam 63931) and rinsing (0.1% TritonX-100 in PBS), sections were incubated for 1 hr with appropriate Alexa-488 or Alexa-594 labeled secondary antibodies (diluted 1∶1000 in blocking buffer). Sections were treated to reduce auto-fluorescence (Autofluorescence Eliminator Reagent, Millipore #2160) and mounted using Mowiol-Dabco containing Hoechst stain (Molecular Probes #33342). All confocal images were acquired using the same settings.

Analysis of myelin was by histological staining (Black Gold II myelin staining [40] (Millipore, AG400) following the provided instructions, and by IHC for 2′,3′-cyclic nucleotide 3′-phosphodiesterase (CNPase) (Millipore, MAB326) and for Myelin Proteolipid Protein (PLP) (AbD Serotec, MCA839G).

PSA-NCAM positive cells in the granular cell layer of the dentate gyrus (Fig. S1) were counted under microscopic observation (x64 magnification) with the experimenter blinded to the identity of the sections, as previously described [41]. Three sections per mouse, spaced over 240 µm, were analyzed and results expressed as the number of PSA-NCAM positive cells per 0.15 mm^2^ of granular cell layer of dentate gyrus (DG). Nectin-3 IHC staining intensity was quantified, with the experimenter blind to the identity of the sections, using grey level optical density (Qwin software; Leica) in three different areas of SLM versus stratum radiatum (SR) (Fig. S1). For transgenic models, results were expressed as the optical density (OD)-ratio of SLM/SR, and averaged for each genotype. For the AAV-injected mice models, the OD-ratio of SLM/SR were computed for both the ipsi and contra lateral sides, and results expressed as the ratio of the ipsi over contra lateral side. Regional brain areas were measured using dedicated software (Qwin, Leica).

### Statistical analysis

Statistical analysis was performed using dedicated software (GraphPad Prism v5.03; San Diego, CA). Data-sets were analyzed either by Student's t-test (unpaired, two-tailed) or ANOVA (one-way or two-way), followed by Dunnett or Bonferroni *post hoc* test as indicated in the figure legends. Statistical significance was defined as p<0.05.

## Results

In this study, we aimed to analyze immunohistochemically and biochemically the expression and localization of selected CAMs (Table 1) in the brains of transgenic mice that are validated models for amyloid and tau pathology [5], [24]–[26], [29], [35], [42], [43]. The CAMs analyzed in this study were selected for their reported or presumed contribution to cognitive brain functions, particularly to deficits in memory and learning as affected by normal ageing and by chronic diseases associated with old age.

**Table 1:** Overview of the Cell Adhesion Molecules analyzed.

CAM	Main Characteristics	Localization (tentative)	References	Antibodies
NCAM	3 isoforms distinguished by MW	Pre- & post-synaptic	[49], [64]–[66]	AbCys AbC0026
	Role in:	NCAM-180 postsynaptic		
	- Development	NCAM-140 neurons & glia		
	- Plasticity	NCAM-120 mainly glia		
	- Cognition			
PSA-NCAM	Polysialylated version of NCAM	Pre- & post-synaptic	[41], [67], [68]	AbCys AbC0019
	Role in:			
	- Spatial memory			
SynCAM	4 isoforms distinguished by MW	Pre- & post-synaptic	[69]–[72]	Sigma S4945
	Role in:			
	- Cognition			
Nectin-1	Interacts with Nectin-3	Puncta adherentia junctions	[12], [14], [36]	gift H.J. Federoff
	Binds actin (l-Afadin)	Pre-synaptic (CA3		
Nectin-3	Interacts with Nectin-1	Puncta adherentia junctions	[12], [14], [60], [61]	AbCam 63931
	Binds actin (l-Afadin)	Post-synaptic (CA3)		
		Axonal		

Abbreviations: MW, molecular weight; NCAM, neural cell adhesion molecule; PSA, polysialic acid.

### NCAM

**Figure 1:**
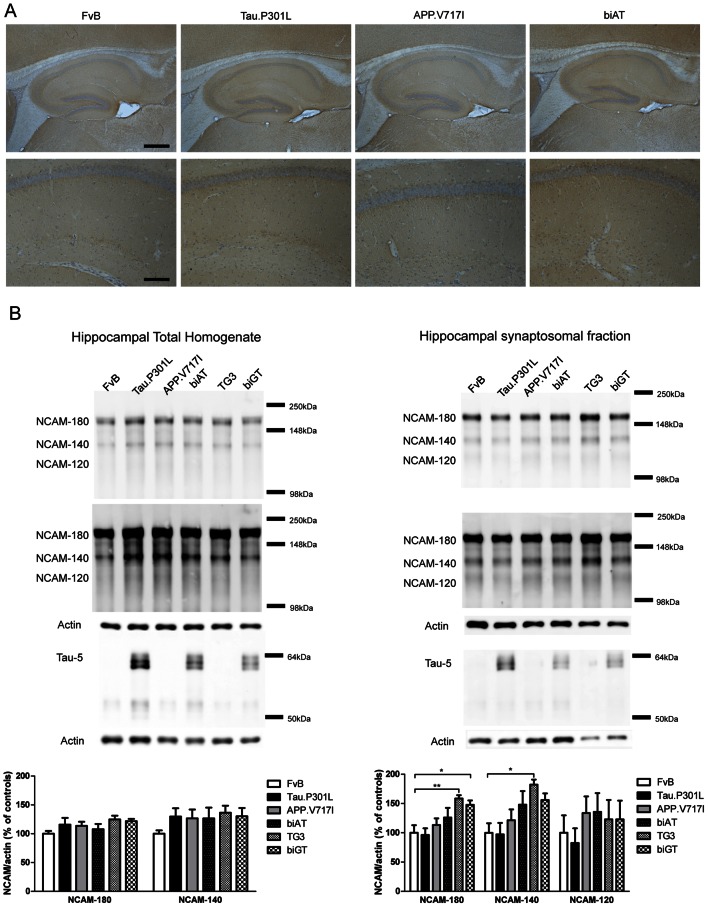
Expression of NCAM in the hippocampus and in synaptosomes. A. NCAM expression in hippocampus (upper panels) and CA1 (lower panels) of selected transgenic mice (age 4 months) compared to wild-type FvB mice. Images are representative of the median expression observed (n = 5/6 per genotype). Scale bars: 400 µm in upper panels, 100 µm in lower panels. B. NCAM expression in total homogenates (left panels) and hippocampal synaptosomes (right panels) (n = 4 per genotype). NCAM expression was normalized to actin levels and presented relative to wild-type FvB mice. Note that NCAM-120 signals were at least one order of magnitude weaker, hampering reliable quantification. Differential exposure times were used for the NCAM-180 isoform and NCAM-140/120 isoforms. The presence of human Tau was verified biochemically in each genotype and preparation tested. Data: mean ± SEM, statistically analyzed by one-way ANOVA followed by Dunnet *post hoc* test with respect to FvB (* p<0.05, ** p<0.01).

NCAM, arguably the best characterized neuronal CAM, presents biochemically as 3 major isoforms: two transmembrane isoforms denoted NCAM-180 and NCAM-140, while NCAM-120 is GPI-anchored [44], [45]. Unfortunately, the lack of isoform-specific antibodies makes it impossible to distinguish between the three isoforms in IHC. Nevertheless, NCAM was observed to be located throughout the brain, most prominently in the CA3 and DG sub-regions of the hippocampus (Fig. 1A, upper panels). NCAM was evident in somata of CA1 pyramidal neurons and their apical dendrites (Fig. 1A, lower panels). Neither the pattern nor the intensity of NCAM expression was consistently altered in the different mice analyzed for each genotype (Fig. 1A).

NCAM was further analyzed biochemically in total hippocampal homogenates and observed mainly as the two larger isoforms, with the 120 kDa isoform much less prominent (Fig. 1B). No significant changes in the levels of NCAM-140 and NCAM-180 were evident in any genotype, relative to wild-type FvB mice (Fig. 1B; one-way ANOVA; NCAM-140 F_(5,18)_ = 0.8239, p = 0.5488 and NCAM-180 F_(5,18)_ = 1.391, p = 0.2742). To measure synaptic NCAM expression more specifically, we prepared hippocampal synaptosomes from all genotypes. In these synaptosomal preparations, NCAM-180 had a significantly higher expression level in the TG3 and biGT mice than wild-type mice (Fig. 1B; one-way ANOVA: F_(5,18)_ = 4.934, p = 0.0051), the two genotypes that express human GSK3β.S9A. A similar change was observed in both these genotypes for the NCAM-140 isoform, but statistical significance was reached only in the TG3 mice, not in the biGT mice (Fig. 1B; one-way ANOVA: F_(5,18)_ = 3.925, p = 0.0139). No significant differences between genotypes were observed for NCAM-120 isoform (Fig. 1B; one-way ANOVA; F_(5,18)_ = 0.475, p = 0.7901).

As an additional control, the total homogenate and synaptosomal preparations from the hippocampi of Tau.P301L mice and in all derived genotypes were checked for tau protein expression. Expression of Tau (Fig. 1B) was similar to that previously reported [35].

### PSA-NCAM

**Figure 2:**
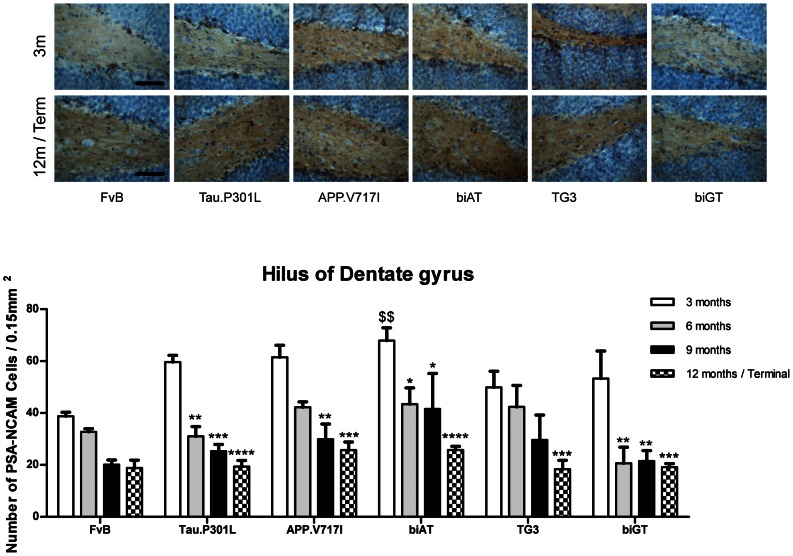
PSA-NCAM expression in the Dentate Gyrus. Upper panel: Representative images of IHC for PSA-NCAM expression in young (3 months) and old (12 month or terminal) mice with genotypes indicated. Scale bar: 50 µm. Lower panel: Number of PSA-NCAM positive neurons in the dentate gyrus of mice aged 3, 6, 9, and 12 months (n = 3 to 6 per age-group). Data: mean ± SEM, analyzed by two-way ANOVA followed by Bonferroni *post hoc* test: *p<0.05, **p<0.01, ***p<0.001, ****p<0.0001 (* relative to 3 months); ^$$^p<0.01 (^$^ relative to FvB).

NCAM is modified post-translationally by addition of α-2,8-linked sialic acid to form polysialylated NCAM (PSA-NCAM) presenting as a separate structural and functional entity. PSA-NCAM is expressed in brain regions with high levels of synaptic plasticity, predominantly in the developing nervous system, but also in adult brain in areas involved in memory and learning, particularly the hippocampus [46]–[50]. In agreement with these and other reports, we observed that PSA-NCAM was expressed mainly on neurons that border the hilar region of the DG (Fig. 2, upper panel).

We measured the number of PSA-NCAM positive neurons in the DG and this was demonstrated to depend on the genotype and also on the age of the mice within each genotype (Fig. 2). Interestingly, at a young age, all transgenic genotypes tended to have higher numbers of PSA-NCAM expressing-neurons in the DG than wild-type mice (Fig. 2). These differences reached statistical significance in young biAT mice compared to age and gender-matched wild-type mice (Fig. 2). Age-dependent decline of PSA-NCAM expression in the DG was evident in all mice models relative to wild-type mice, independent of the genotype (Fig. 2; two-way ANOVA: genotype: F_(5,76)_ = 5.515, p = 0.0002; age: F_(3,76)_ = 44.16, p<0.0001; interaction: F_(15,76)_ = 0.9852, p = 0.4782). As these observations are consistent with the usual age-related changes in PSA-NCAM reported, this suggests that the contribution of this specialized CAM to defects resulting from ageing may not be specifically related to those inflicted by amyloid or tau pathology.

### SynCAM

**Figure 3:**
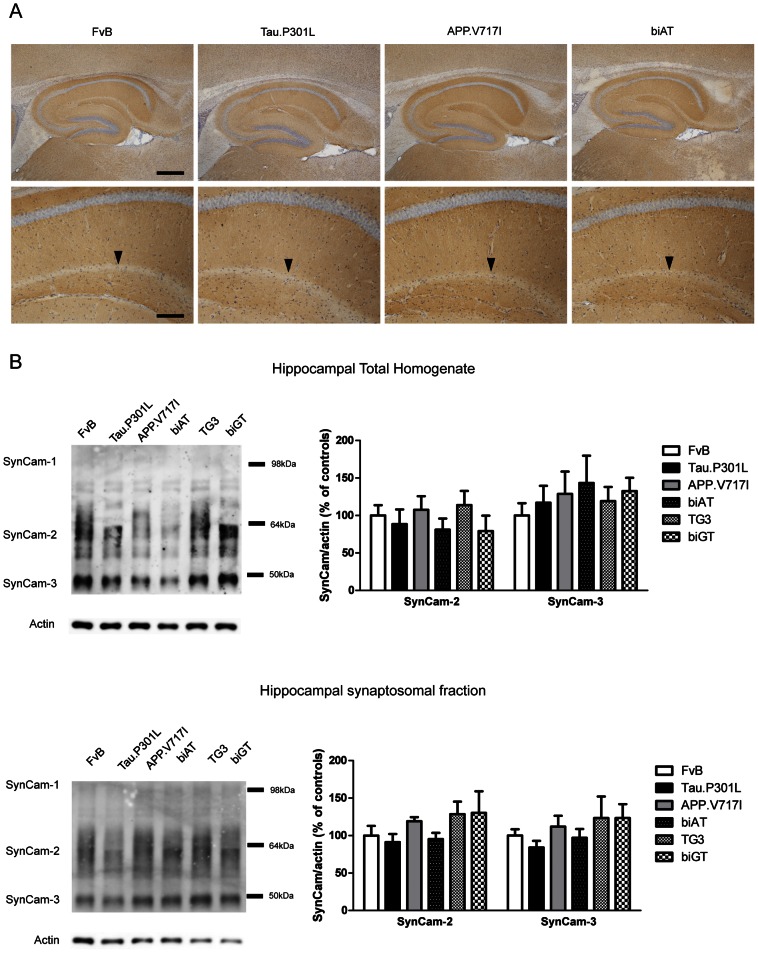
SynCAM in the hippocampus and synaptosomes. A. IHC for SynCAM in hippocampus (upper panels) and CA1 region (lower panels) of young mice (age 4 months) of the indicated genotypes. Arrowheads denote the layer between CA1 SLM and SR in which SynCAM is absent. Images are representative for median expression observed (n = 5/6 per genotype). Scale bars: 400 µm in upper panels, 100 µm in lower panels. B. Biochemical analysis of SynCAM isoforms in total homogenates (upper panels) and synaptosomal preparations (lower panels) from the hippocampus (n = 4 per genotype). Data were normalized to actin levels and reported relative to those in wild-type FvB mice. Quantification of SynCAM-1 was not possible because of the poor reaction with the antibody, producing too weak signals. Data: mean ± SEM, analyzed by one-way ANOVA followed by Dunnett *post hoc* test with respect to FvB.

IHC showed SynCAM-1 to be strongly expressed within the hippocampus (Fig. 3A, upper panel). Particularly, the CA1 region, including the SLM, stained intensely for SynCAM-1, although a marked zone along the border of the SLM with the stratum radiatum (SR) remained nearly devoid of SynCAM-1 (Fig. 3A, lower panels, arrowheads). Similar patterns were observed in all mice, independent of their genotype by IHC staining for SynCAM-1 (Fig. 3A).

Biochemical analysis of SynCAM-1 by western blotting revealed multiple protein species reacting with the same antibody (S4945) used for IHC. Although this antibody is reported to be specific for SynCAM-1, we consistently observed additional SynCAM isoforms (Fig. 3B, upper left-hand panel). As the SynCAM-1 (100 kDa), -2 (60–75 kDa) and -3 (48 kDa) isoforms were clearly distinguished by western blotting, as previously reported by others [51], we consider the S4945 antibody to be a pan-SynCAM antibody.

We analyzed the different SynCAM isoforms biochemically in total homogenates and in hippocampal synaptosomes prepared from all genotypes. The levels of the two smaller SynCAM isoforms did not differ significantly in total hippocampal homogenates (Fig. 3B; one-way ANOVA: SynCAM-2, F_(5,18)_ = 0.6476, p = 0.6669 and SynCAM-3, F_(5,18)_ = 0.3669, p = 0.8645) nor in hippocampal synaptosomal preparations from the different genotypes (Fig. 3B; one-way ANOVA: SynCAM-2, F_(5,18)_ = 1.202, p = 0.3476 and SynCAM-3, F_(5,18)_ = 0.8915, p = 0.5073). Quantification of the 100 kDa SynCAM-1 isoform was hampered by the much stronger chemiluminescence signals from the bands representing SynCAM-2 and SynCAM-3, which interfered with the analysis of SynCAM-1.

### Nectins

**Figure 4:**
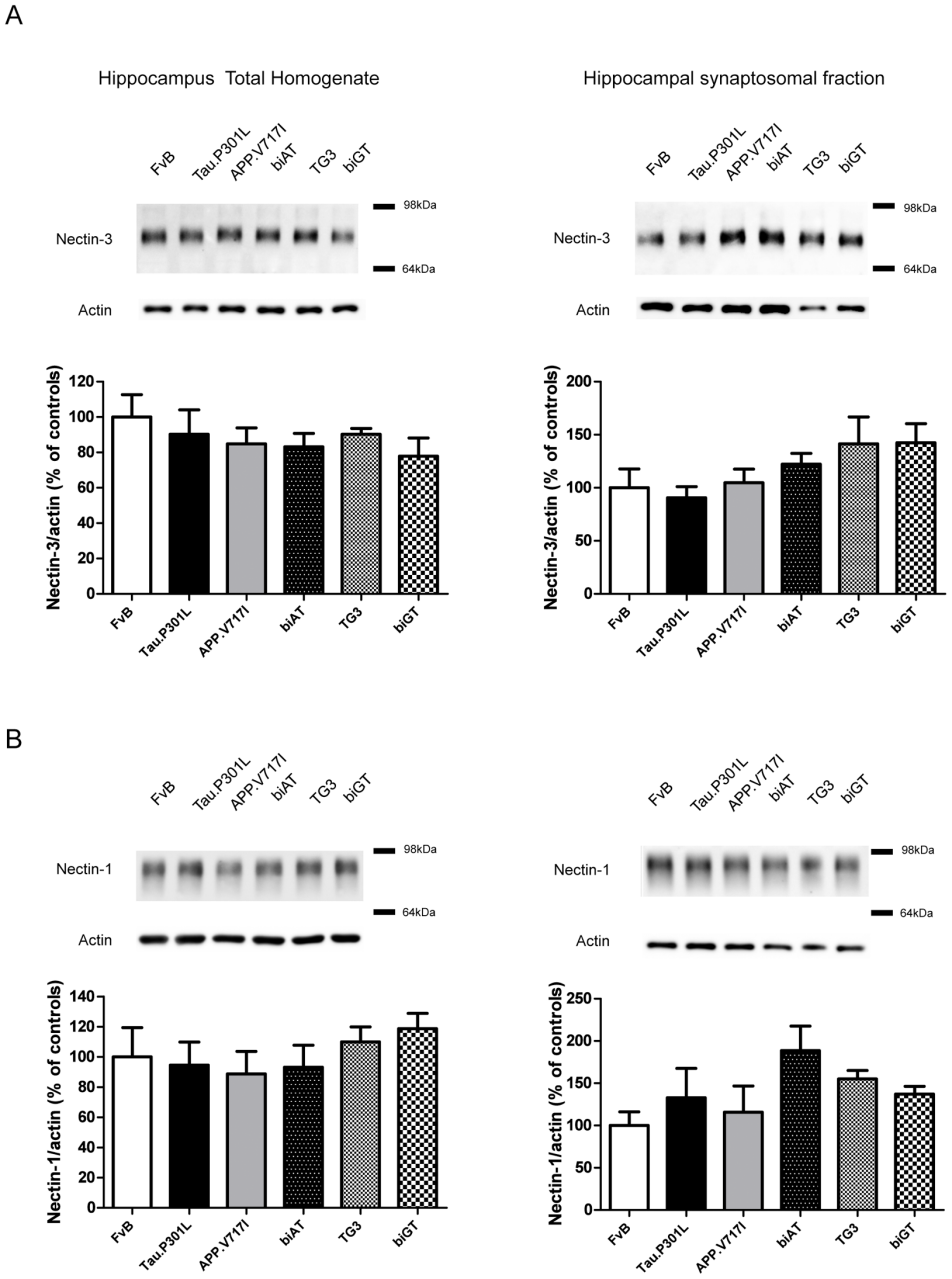
Nectin-3 in the hippocampus and synaptosomes. Nectin-3 levels in total homogenates and synaptosomal preparations from hippocampus (n = 4 per genotype) normalized to actin levels and expressed relative to levels in wild-type FvB mice. Data: mean ± SEM, analyzed by one-way ANOVA followed by Dunnett *post hoc* test with respect to FvB.

**Figure 5:**
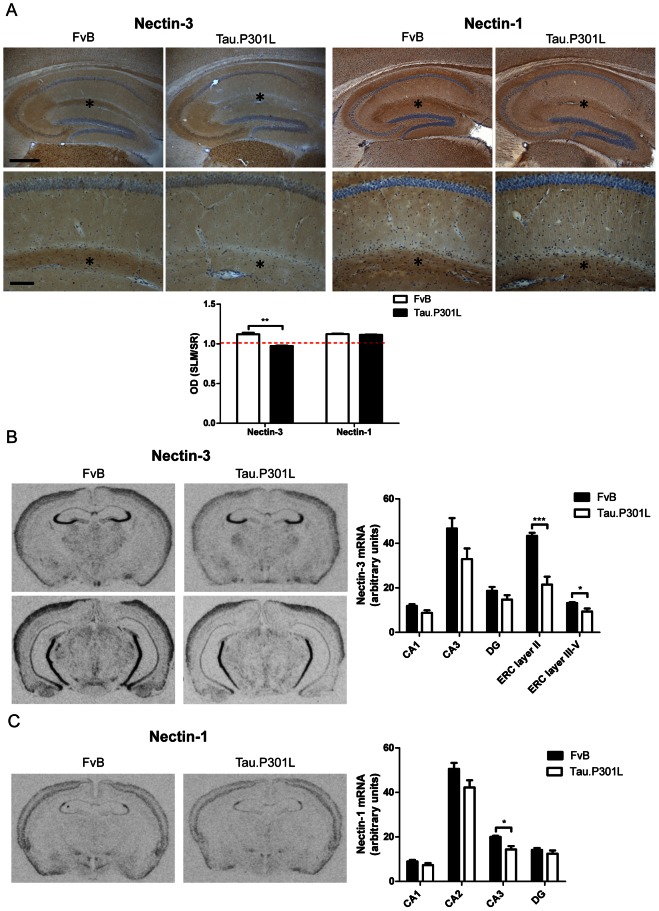
Nectin-1 and Nectin-3 protein and mRNA expression. A. IHC for Nectin-3 and Nectin-1 proteins in hippocampus (upper panels) and CA1 (lower panels) of Tau.P301L and wild-type FvB mice. Images are representative for the median of expression observed (n = 3). Levels of Nectin-3 and Nectin-1 are expressed as the ratio of optical densities measured in SLM and SR of CA1. The red broken line (ratio = 1) is added to illustrate the relative levels of Nectin-3 in SLM versus SR, emphasizing the decrease of Nectin-3 in CA1 SLM. Data are represented as mean ± SEM (n = 3 per genotype), analyzed by unpaired Student's t-test (two-tailed); ** p<0.01. Scale bars: 400 µm in upper panels, 100 µm in lower panels. Asterisks denote the CA1 SLM region. B. In situ hybridization for Nectin-3 mRNA in the brain of Tau.P301L and wild-type FvB mice. Data: mean ± SEM (n = 8 and 7, respectively), analyzed by unpaired Student's t-test (two-tailed). C. In situ hybridization for Nectin-1 mRNA in the brain of Tau.P301L and wild-type FvB mice. Values reported are mean ± SEM (n = 8 and 7, respectively), analyzed by unpaired Student's t-test (two-tailed)); * p<0.05, *** p = 0.001. C. In situ hybridization for Nectin-1 mRNA in the brain of Tau.P301L and wild-type FvB mice. Values reported are mean ± SEM (n = 8 and 7, respectively), statistically analyzed by unpaired Student's t-test (two-tailed); * p<0.05.

Biochemical analysis of total hippocampal homogenates and of hippocampal synaptosomes demonstrated that the overall levels of Nectin-3 were not markedly affected in the transgenic models (Fig. 4A; one-way ANOVA: total homogenates F_(5,18)_ = 0.5732, p = 0.7198 and synaptosomes F_(5,18)_ = 1.734, p = 0.1779). Similarly, biochemical analysis of hippocampal total extracts or synaptosomal fractions did not show any effects on Nectin-1 protein levels in any of the genotypes (Fig. 4B; one-way ANOVA: total homogenates F_(5,18)_ = 0.6277, p = 0.6809 and synaptosomes F_(5,18)_ = 1.671, p = 0.1926). IHC demonstrated Nectin-3 expression in the hippocampus of wild-type mice predominantly in the CA3 and the SLM of CA1 (Fig. 5A). Unexpectedly, a marked decrease, or even absence of Nectin-3 was observed in the CA1 SLM of Tau.P301L mice (Fig. 5A; t-test: p = 0.0022). In contrast, again Nectin-1 was not affected in the SLM of Tau.P301L mice (Fig. 5A; t-test: p = 0.3622). We concluded that the observed under-representation of Nectin-3 in the SLM of Tau.P301L mice was not a general defect, but specific for this specialized CAM.

### Nectin-3 defect in SLM of Tau.P301L mice

The SLM of CA1 acts as a connection hub for the ERC to hippocampus, linked by the temporoammonic pathway (TA). The ERC and CA regions are the first limbic structures targeted by the pathology of AD, before it spreads to other brain regions [18], [19], [52]. To determine whether the decreased levels of Nectin-3 in the SLM of Tau.P301L mice originated in the CA1 or in the ERC, we performed in situ hybridization to detect the mRNA of both Nectins.

Nectin-3 mRNA was most prominent in the hippocampal CA subregions (Fig. 5B, left panels) and tended to be lower in CA sub-regions of Tau.P301L mice, but the difference was not statistically significant relative to wild-type mice (Fig. 5B; t-test: CA1, p = 0.0530; CA3, p = 0.0692). In contrast, the marked decrease of Nectin-3 mRNA was evident in the ERC, particularly in layer II (Fig. 5B; t-test: layer II, p = 0.001; layer III-V, p = 0.0205). This suggested two possible causes for the low levels of Nectin-3 protein in SLM: either the low levels of mRNA indicate an impaired synthesis of Nectin-3 by ERC neurons, or more likely the impaired transport of Nectin-3 protein from ERC to SLM in Tau.P301L mice.

The levels of Nectin-1 mRNA were decreased in the CA3 of Tau.P301L mice relative to wild-type mice (Fig. 5C; t-test: CA3, p = 0.0101). The decrease was nevertheless less pronounced than that of Nectin-3 in the ERC, which may explain why Nectin-1 protein levels were not markedly affected in the hippocampus of Tau.P301L mice.

**Figure 6:**
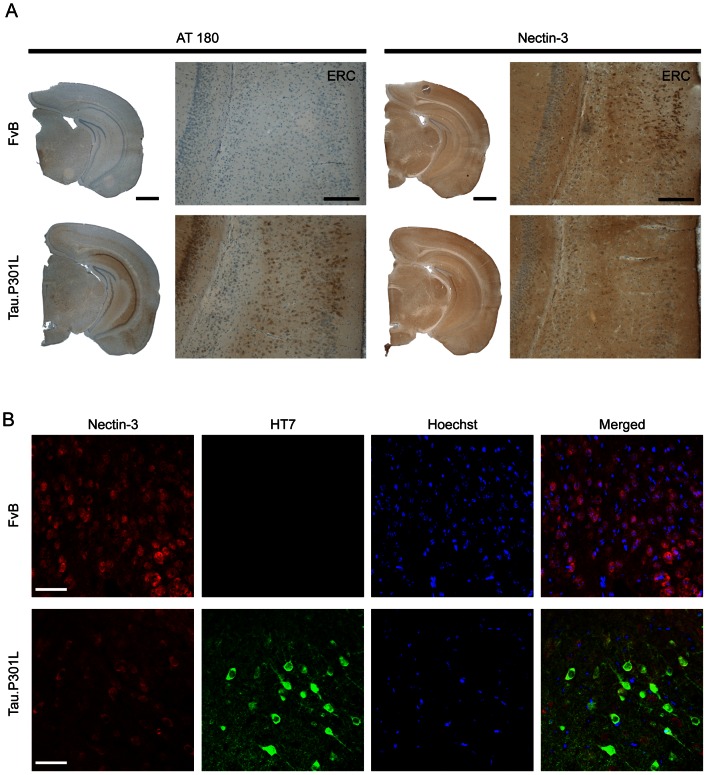
Nectin-3 T231-Tau in entorhinal cortex. A. IHC for pT231-Tau (AT180) and Nectin-3 in the entorhinal cortex of wild-type FvB mice and Tau.P301L mice. Images are representative of the median expression observed (n = 3). Scale bars: 1 mm in upper panels, 200 µm in lower panels. B. IHC for total human protein tau (HT7; green), Nectin-3 (red) and Hoechst (blue) in the entorhinal cortex of wild-type FvB mice and in Tau.P301L mice. Images are representative of the median expression observed (n = 4). Scale bars: 50 µm.

We analyzed whether expression of protein Tau itself was affected in the SLM of Tau.P301L mice, and compared by IHC the localization of Nectin-3 to that of selected phosphorylated Tau isoforms. Among the different phospho-specific antibodies analyzed, AT180, defining the pT231 phospho-epitope on protein Tau, proved most informative. AT180 markedly labeled neurons and processes in the hippocampus, as well as in ERC layers II to V of Tau.P301L mice (Fig. 6A). Moreover, on serial sections, the same neurons and layers of the ERC were observed to have a lower intensity of staining and lesser density of Nectin-3 positive neurons in the ERC of Tau.P301L mice, relative to wild-type mice (Fig. 6A).

Moreover, neurons expressing human protein Tau in the ERC of Tau.P301L mice, stained much weaker for Nectin-3 (Fig. 6B), resulting in less cells showing colocalization. Moreover, the images clearly illustrated the important decreased number of neurons that expresses Nectin-3 in Tau.P301L mice, compared to wild-type FvB mice.

These combined data therefore suggested that expression of mutant Tau.P301L, and the concomitant early stages of beginning tauopathy marked by increased phosphorylation of protein tau in the entorhinal cortex, specifically impairs the expression of the synaptic CAM Nectin-3 in the SLM of the hippocampus.

### Nectin-3 defect in SLM of other transgenic models

**Figure 7:**
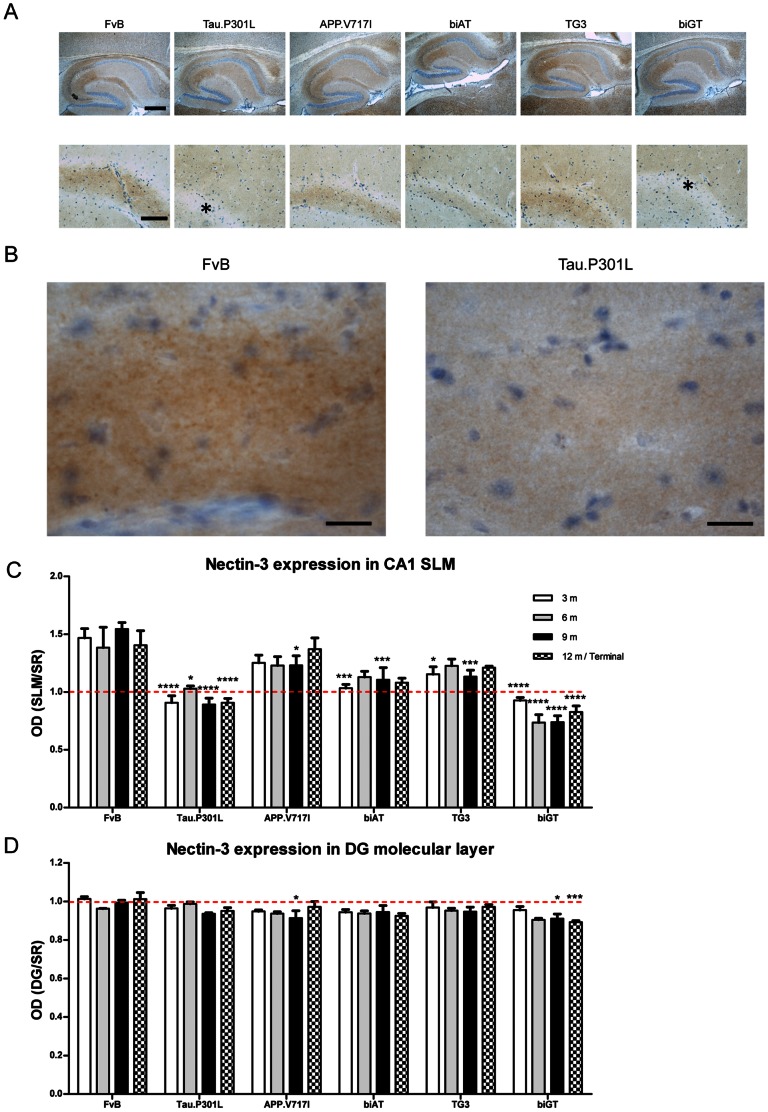
Expression of Nectin-3 in the SLM. A. IHC for Nectin-3 in hippocampus and CA1 of young mice with the genotypes indicated (age 3 months). Asterisks denote low or negative Nectin-3 expression within SLM of CA1. Scale bars: 400 µm in upper panels, 100 µm in lower panels. B. Representative higher power images of SLM of FvB and Tau.P301L mice. Scale bar: 20 µm. C. Levels of Nectin-3 in mice with the indicated genotypes at age 3, 6, 9 and 12 months or Terminal (n = 3 to 6). Expression levels are the ratio of optical densities measured in SLM and SR of CA1. The red broken line (at ratio = 1) represents the relative levels of Nectin-3 in SLM versus SR, is included to illustrate the relative decrease of Nectin-3 in SLM in Tau.P301L and biGT mice at all ages. Data, mean ± SEM, analyzed by two-way ANOVA followed by Bonferroni *post hoc* test when appropriate (* p<0.05, ** p<0.01, *** p<0.001, ****p<0.0001, relative to wild-Type FvB mice). D. Levels of Nectin-3 in the molecular layer (ml) of the DG relative to SR in the different genotypes at age 3, 6, 9 and 12 months or Terminal (n = 3 to 6). The red broken (ratio = 1) delineates equal levels of expression of Nectin-3 in the ml layer of the DG and in the CA1 SR. Data are represented as mean ± SEM, statistically analyzed by two-way ANOVA followed by Bonferroni *post hoc* test when appropriate (* p<0.05, *** p<0.001, relative to wild-type FvB mice).

The specific defect in the expression of Nectin-3 in the SLM of Tau.P301L mice led us to investigate whether Nectin-3 expression was affected in our other models for amyloid and tauopathy. Interestingly, the outcome of IHC analysis was clear-cut: Nectin-3 protein was not only significantly reduced in the monogenic Tau.P301L mice, but also in the bigenic biGT mice, combining Tau.P301L x GSK3β.S9A transgenes, without any effect of age (Fig. 7A, B, C; two-way ANOVA: genotype: F_(5,76)_ = 45.53, p<0.0001; age: F_(3,76)_ = 0.1511, p = 0.9287; interaction: F_(15,76)_ = 1.085, p = 0.3844, upper panel*). Moreover, a marked reduction of Nectin-3 levels in the SLM was also observed in transgenic mice that express human wild-type Tau.4R protein [31], [32], while mice deficient in endogenous mouse protein Tau had normal expression levels of Nectin-3 in their SLM (data not shown). Interestingly, biAT mice that express human Tau.P301L in combination with human APP.V717I transgenes, and thereby develop the combined amyloid and tau pathology typical for AD [26], also showed reduced Nectin-3 levels in the SLM (Fig. 7A, C). In contrast, the parental APP.V717I mice, with amyloid pathology only, presented with unaltered Nectin-3 levels in the SLM (Fig. 7A, C).

Our combined results corroborate the hypothesis that a reduction in Nectin-3 expression in the hippocampal SLM is specifically induced by expression of human protein tau. The observation that expression of human wild-type Tau.4R and human mutant Tau.P301L protein led to the same defect in Nectin-3 in the SLM, suggested a similar underlying mechanism. Nevertheless, the phenotypes of Tau.4R and Tau.P301L mice differ specifically: severe axonopathy in the Tau.4R mice caused by microtubuli-associated transport problems is not observed in Tau.P301L mice [25], [26], [31], [32], [53].

Because Nectin-3 expression was affected by protein Tau in CA1 SLM, which contains the TA projections, we also investigated Nectin-3 expression within the molecular layer of the DG, which is the perforant path area of projection. Interestingly, Nectin-3 levels in the molecular layer of the DG were significantly affected by the genotype (Fig. 7D; two-way ANOVA: genotype: F_(5,76)_ = 8.078, p<0.0001; age: F_(3,76)_ = 1.979, p = 0.1242; interaction: F_(15,76)_ = 1.160, p = 0.3208). Of note, however, only in old Tau.P301L mice (age 9 months) tended to decrease Nectin-3, whereas in old biGT mice the reduction was significant, with a trend already at young age (3 months).

### Nectin-3 defect in sub-acute AAV-Tau models

The specific defect in Nectin-3 expression in the SLM that we consistently observed was in three strains of transgenic mice that express human protein tau postnatally (weeks 2–3) as instructed by the mouse Thy1-gene promoter. This led us to question whether the same defect would occur in the sub-acute Tauopathy model that we recently developed, based on intracerebral injection of recombinant AAV vectors [5], [27], [43].

**Figure 8:**
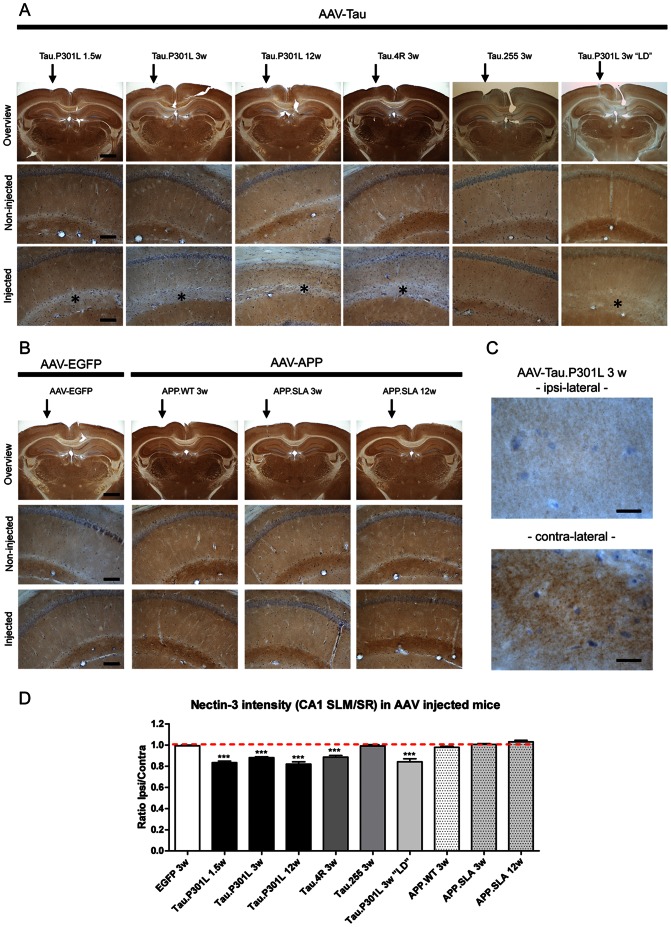
Nectin-3 in sub-acute AAV-based models. A. IHC for Nectin-3 in brain of wild-type mice following intracerebral injection of AAV-Tau vectors, comparing ipsi-lateral (arrow) to contra-lateral hemisphere, analyzed 1.5 to 12 weeks post injection as indicated. Mice injected with lower dose (LD, panels at right) of AAV-Tau.P301L did not show neurodegeneration [5], [27] and were included to demonstrate that expression Tau and not neurodegeneration causes the decrease in Nectin-3 in SLM (denoted by asterisks). Representative images for median expression (n = 4). Scale bars: 1 mm in upper panels, 100 µm in middle and lower panels. B. IHC for Nectin-3 in brain of wild-type mice after intracerebral injection of specified AAV-vectors, analyzed 3 to 12 weeks post injection, as controls to demonstrates lack of effect of the AAV virus (AAV-EGFP) and of AAV-APP on Nectin-3 in SLM. Representative images for median expression (n = 4). Scale bars: 1 mm in upper panels, 100 µm in middle and lower panels. C. IHC for Nectin-3 in SLM of AAV-Tau.P301L injected wild-type mice (3 weeks post-injection) at higher magnification. Scale bars: 20 µm. D. Ratio of optical densities of IHC for Nectin-3 in CA1 SLM and SR in ipsi- and contra-lateral hemispheres for the different AAV-Tau and AAV-APP vectors injected in wild-type mice (n = 4, except AAV-APP.SLA 12 weeks n = 3). Data: mean ± SEM, analyzed by one-way ANOVA followed by Dunnett *post hoc* test with respect to AAV-EGFP as control (***p<0.001).

Following intracerebral injection of AAV-Tau.P301L in the hippocampus of wild-type mice, a marked decrease in Nectin-3 protein expression was already evident at 10 days post-injection, prior to signs of neurodegeneration (Fig. 8A, D; one-way ANOVA: F_(9,29)_ = 27.31, p<0.0001). Subsequent analysis after injection of other AAV-vectors, and at later time-points post-injection, not only confirmed the localized Nectin-3 protein decrease in the SLM by the AAV-Tau.P301L vector (Fig. 8A, C, D) but also by the AAV-Tau.4R vector that confers expression of wild-type human Tau.4R (Fig. 8A, D). In contrast, similar intracerebral injection in wild type mice of AAV-Tau.255, encoding the C-truncated version of protein tau that is devoid of the C-terminal microtubule-binding domains [5], [27], [43], did not affect Nectin-3 levels in SLM (Fig. 8A, D). These combined observations provided additional conclusive evidence for a direct, inverse relation between the expression of human wild-type Tau.4R or mutant Tau.P301L and the CAM Nectin-3 within the SLM. Moreover, injections of lesser doses of AAV-Tau vectors did reduce or even eliminate the neurodegeneration in CA1, but still caused the reduction of Nectin-3 in the SLM of CA1 (Fig. 8A, D).

Of note, similar intracerebral injections in wild-type mice of either AAV-APP or AAV-APP.SLA to express human wild-type APP or triple mutant APP.SLA [5], [27], [43] did not affect the expression of Nectin-3 within the SLM, not even at 3 months post-injection (Fig. 8B, D). As an additional control, we injected AAV-EGFP vectors intracerebrally in wild-type mice and observed normal levels of Nectin-3 in the SLM (Fig. 8B, D). The results support and extend our observations on Nectin-3 expression in the APP.V717I and biAT transgenic mice and exclude amyloid-related effects on Nectin-3 expression in the SLM.

Finally, injection of AAV-Tau.4R in the ERC led to strong expression of human Tau in the TA and PP pathways (Fig. S2). Moreover, the concomitant selective decreased expression of Nectin-3 in CA1 SLM underscored the hypothesis that at least a major part of Nectin-3 protein originated in ERC neurons.

We conclude that neurodegeneration of CA1 pyramidal neurons in AAV-Tau models does not explain the lack of Nectin-3 protein in the CA1 SLM and that the origin of Nectin-3 in SLM is most likely located in the ERC. This outcome fits the accepted localization of Nectin-3 as mainly axonal, although some dendritic contribution cannot be excluded. These conclusions would further imply that Nectin-3 mRNA or protein is transported from the ERC to the SLM, by way of the TA, resulting in the localization of Nectin-3 in the pre-synaptic compartments of the TA-axons in the SLM.

## Discussion

Cognitive decline, late in normal ageing and early in neurodegenerative disorders, must find its causes at the level of dysfunctional synapses that lead only later, directly or indirectly, to degeneration of the neurons they connect. The brain-regions that are affected by normal ageing and/or compromised by disease vary considerably individually, depending on unknown genetic and epigenetic factors. In neurodegenerative diseases, the affected brain regions are generally well delineated, for instance the hippocampal formation in AD, or the frontal lobe in fronto-temporal dementia. In addition to the regional diversity, the structural and functional composition of synapses varies in different brain-regions, posing additional analytical problems and interpretation issues. Among the many proteins that are needed to make up functional synapses, CAMs are important partners. Many different types of CAMs are known as integral synaptic constituents, needed for the structure but also for the plasticity of synapses, making CAMs subject to change for physiological adaptation, but also vulnerable in disease processes.

Our combined data argued neither for a general nor for a consistent effect on the different synaptic CAMs in the transgenic mouse models analyzed. Our studies showed widely differing responses: some CAMs were not affected, while others were increased or decreased in specified brain-regions. NCAM, PSA-NCAM and SynCAM were demonstrated to be either marginally affected, or to be not informative for the specific processes that we are interested in. Interestingly, the demonstrated effects of GSK3β on NCAM may relate to the phosphorylation of NCAM by this important kinase [54], while conversely, NCAM-derived peptides modulate GSK3β-medicated intracellular signaling [55]. Of interest, young transgenic mice of all genotypes tended to express higher levels of PSA-NCAM than wild-type mice in the hippocampus, but all underwent a similar age-dependent decline in PSA-NCAM, as documented previously for wild-type animals [41], [48].

Our most marked, and unexpected finding, was the diminished expression of Nectin-3 in the SLM of all mice that express human protein tau, either wild-type or mutant, and either chronically by transgenesis or sub-acutely by intracerebral AAV-vector injection. Unlike SynCAM and NCAM that are located within the synaptic cleft, Nectins are predominantly found at puncta adherentia junctions. Nectin-3 has been proposed to be asymmetrically distributed, and to differ from Nectin-1 respectively by post- and pre-synaptic locations, presumed to allow heterologous interaction [12]. Interestingly, the decrease in Nectin-3 was already apparent early in the disease process, long before the appearance of axonopathy or fibrillar intra-neuronal tauopathy in the transgenic models, or of CA1 neurodegeneration in the AAV-models [5], [25]–[27], [31], [32].

All data obtained in the different transgenic genotypes that express human protein Tau chronically, in combination with the acute models of AAV-Tau vectors injected intracerebrally, converge on, and support the conclusions.

The effect on expression of Nectin-3 in the SLM by the beginning tauopathy, prior to Tau aggregation, was both rapid and early. Moreover, it is observed only for this CAM and specifically in the SLM, the brain region targeted by myelinated axons originating in the ERC. Interestingly, the pathological process of neurodegeneration and the loss of Nectin-3 was provoked by intracerebral injection of AAV-Tau.P301L and AAV-Tau.4R vectors, but not by AAV-Tau.255 [5], [27], [43]. Obviously, the C-terminal microtubule-binding domain of protein tau is essential to provoke both neurodegeneration and disruption of Nectin-3 expression in the SLM. These data on truncated Tau lacking its microtubular binding and transport module, complement the in situ hybridization results for Nectin-3 mRNA and implicate the microtubular domain of protein tau in the proposed mechanisms: besides the decreased Nectin-3 mRNA levels, we propose disturbed transport from ERC to hippocampus by protein tau to be co-responsible for the reduced expression of Nectin-3 in the SLM.

Our data support the hypothesis that Nectin-3 in the SLM originates from the entorhinal cortex and is transported by the myelinated axons of the temporoammonic pathway [56], [57]. The specific hippocampal region concerned, the SLM of CA1, is of considerable functional interest because of its normal role as the connection hub of ERC to hippocampus. The TA consists of myelinated axons that originate in ERC layer III and synapse on distal parts of the apical dendrites of pyramidal CA1 neurons [57]–[59]. The involvement of these regions is of interest physiologically because of their function in memory and learning. Moreover, these two brain regions also take up center-stage in Alzheimer's disease, in terms of the initiation and the spreading of the pathology [16]–[19], [52].

Although Nectin-3 expression within the molecular layer of the DG region followed a similar trend to decrease, it was far less pronounced. A possible explanation would be the organization of both pathways, illustrated by histological and immunohistological staining for myelin (Black Gold II, CNPase, PLP) (Fig. S3). The TA located within the SLM appears far more dense than the perforant pathway. We continue to analyze structural and functional characteristics of the SLM, in terms of myelinated axons and their synapses, in these mouse models for AD.

The exact consequences for the function of synapses in the SLM need to be defined, but can be predicted to be important. The function of Nectin-3 in the SLM, which according to our hypothesis must be located pre-synaptically, should now be subject to further analysis of eventual functional deficiency inflicted by protein Tau and tauopathy. The proposed pre-synaptic localization of Nectin-3 contrasts with its previously reported post-synaptic localization [14], but supports the axonal localization observed in central and peripheral nervous systems [60], [61].

The SLM undergoes important pathological changes in AD, denoted as “dendritic amputation” [52], which combined with inflammation [62] was proposed to relate to the spatiotemporal spreading of tauopathy in diseased brain [19]. The most recent observations of close correlation between decreased size of SR and SLM with cognitive decline in AD [63] is one additional strong argument to promote a detailed investigation into the functional changes associated with temporoammonic vulnerability in tauopathies. The mouse models presented and characterized here offer essential insights and experimental entry into these problems.

## Supporting Information

Figure S1 **Structures of Hippocampus and entorhinal cortex analyzed.** Left panel: main structures of interest analyzed in this study, with Cornus Ammonis (CA) subfields delimited. CA1 is divided in stratum radiatum (SR) and stratum lacunosum moleculare (SLM). Dentate gyrus (DG) is divided in molecular layer (ml) and granular layer (gl). Right panel: higher magnification of the entorhinal cortex with the different layers of interest numbered from I to VI. Scale bars: 200 µm.
(TIF)

Figure S2 **Intracerebral injection of AAV-Tau.4R in the entorhinal cortex.** A. IHC for total human tau HT7, Nectin-3 and Nectin-1 in wild-type FvB mice injected with AAV-Tau.4R in the entorhinal cortex at coordinates posterior 1.94 mm, lateral 1.4 mm, ventral 2.2 mm relative to bregma. representative images are shown of the hippocampus and its subregions, stained for total human Tau, Nectin-3 and Nectin-1. Scale bars: overview, 400 µm; magnification, 100 µm. B. Ratio of optical densities of IHC for Nectin-3 and Nectin-1 in CA1 SLM versus SR, and in molecular layer of the DG versus CA1 SR (3 sections/mouse).
(TIF)

Figure S3 **Histology and IHC of Myelin.** Histological staining using Black Gold II, and IHC for CNPase and PLP of hippocampus (upper panels) and higher magnifications of TA (*) and PP (+) in respectively CA1 SLM and molecular layer of the DG (lower panels). Scale bars: upper panels:400 µm; lower panels: 100 µm.
(TIF)
