# Supplementary figures and images for: Tauopathy Differentially Affects Cell Adhesion Molecules in Mouse Brain: Early Down-Regulation of Nectin-3 in Stratum Lacunosum Moleculare
Source: PLoS One. 2013 May 21;8(5):e63589. doi: 10.1371/journal.pone.0063589 (PMC3660566; doi:10.1371/journal.pone.0063589)

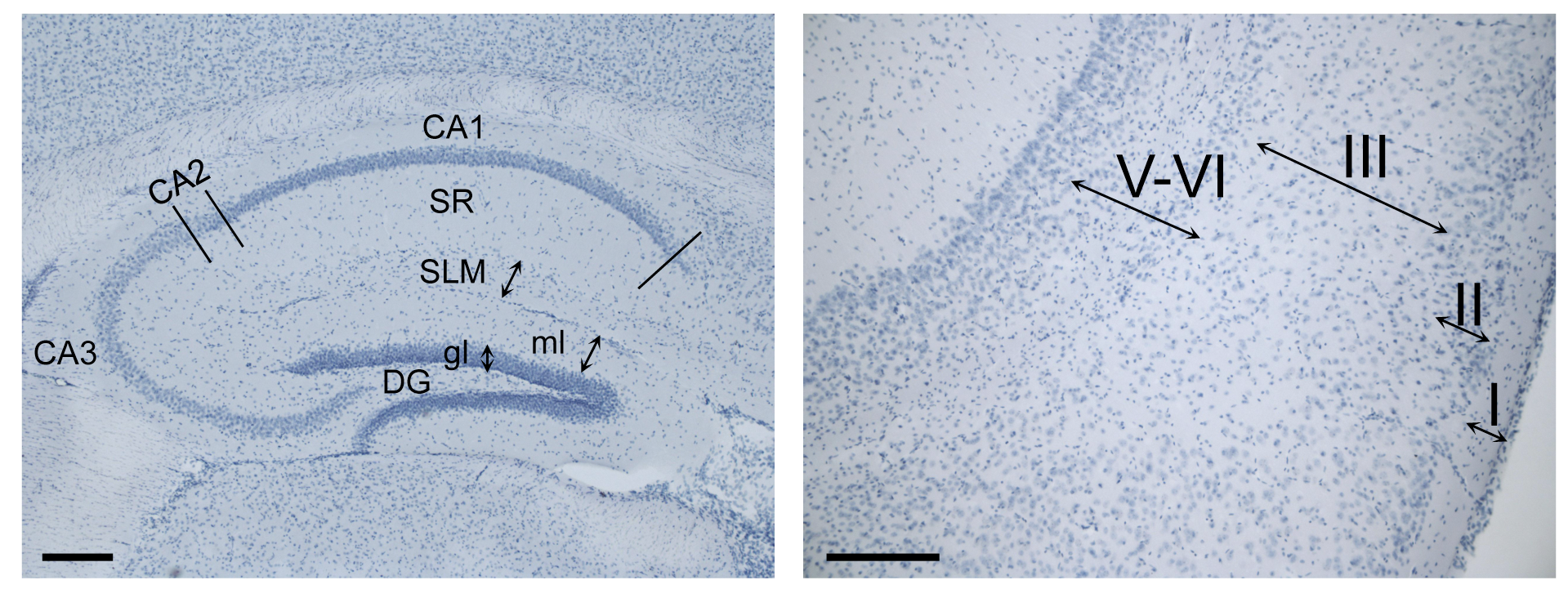

Supplement: Figure S1 — Structures of Hippocampus and entorhinal cortex analyzed. Left panel: main structures of interest analyzed in this study, with Cornus Ammonis (CA) subfields delimited. CA1 is divided in stratum radiatum (SR) and stratum lacunosum moleculare (SLM). Dentate gyrus (DG) is divided in molecular layer (ml) and granular layer (gl). Right panel: higher magnification of the entorhinal cortex with the different layers of interest numbered from I to VI. Scale bars: 200 µm. (TIF) [file pone.0063589.s001.tif]

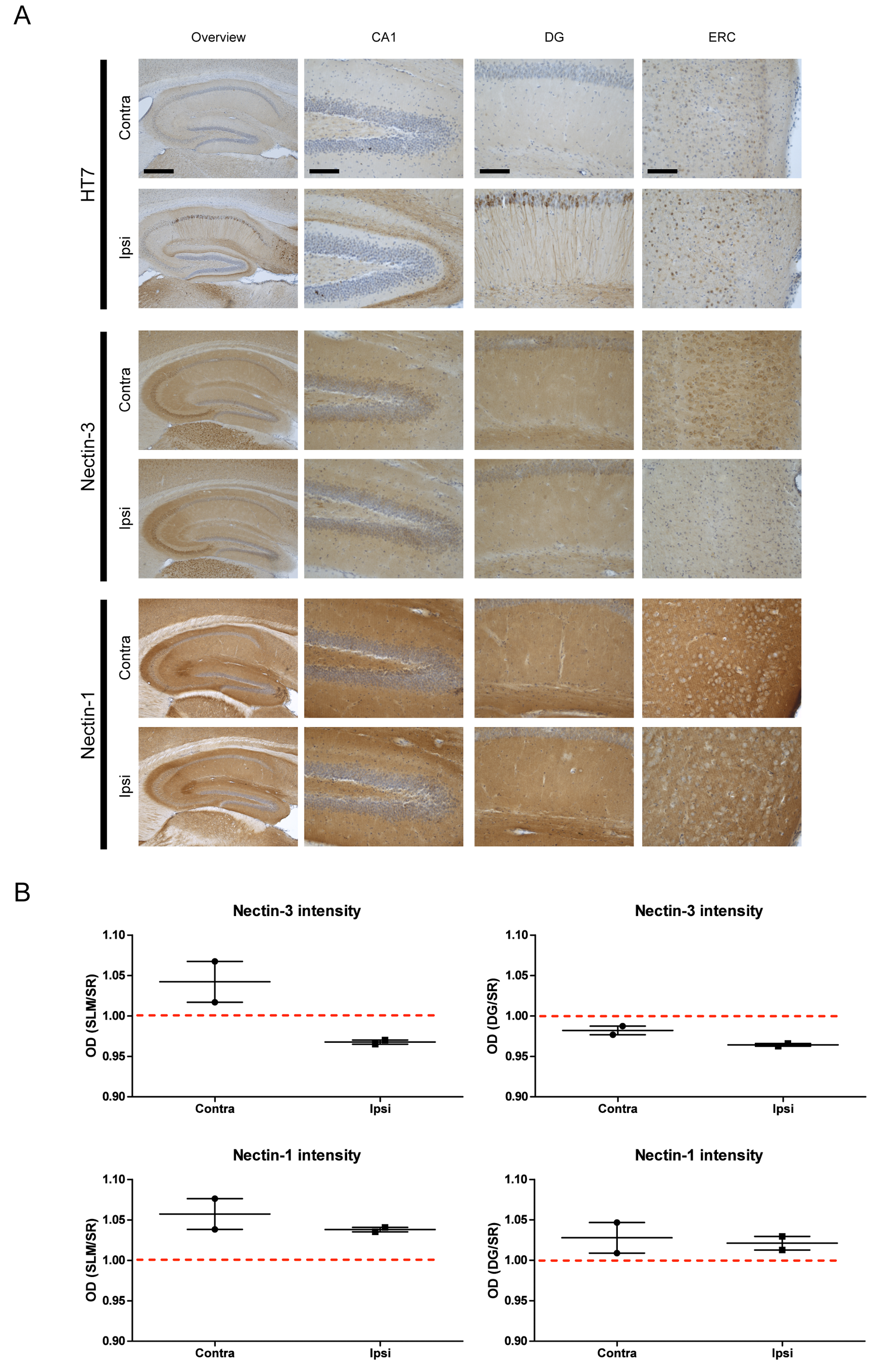

Supplement: Figure S2 — Intracerebral injection of AAV-Tau.4R in the entorhinal cortex. A. IHC for total human tau HT7, Nectin-3 and Nectin-1 in wild-type FvB mice injected with AAV-Tau.4R in the entorhinal cortex at coordinates posterior 1.94 mm, lateral 1.4 mm, ventral 2.2 mm relative to bregma. representative images are shown of the hippocampus and its subregions, stained for total human Tau, Nectin-3 and Nectin-1. Scale bars: overview, 400 µm; magnification, 100 µm. B. Ratio of optical densities of IHC for Nectin-3 and Nectin-1 in CA1 SLM versus SR, and in molecular layer of the DG versus CA1 SR (3 sections/mouse). (TIF) [file pone.0063589.s002.tif]

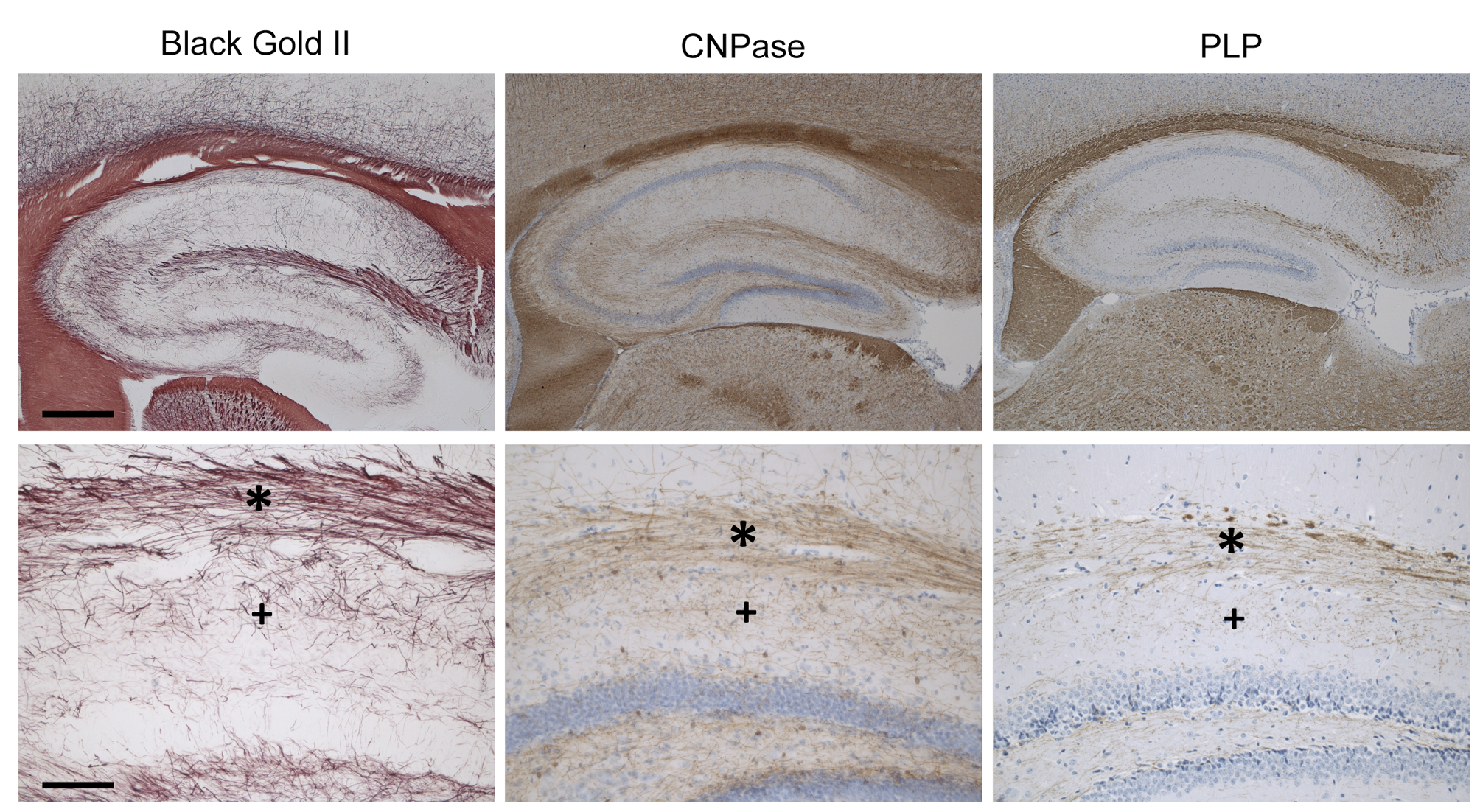

Supplement: Figure S3 — Histology and IHC of Myelin. Histological staining using Black Gold II, and IHC for CNPase and PLP of hippocampus (upper panels) and higher magnifications of TA (*) and PP (+) in respectively CA1 SLM and molecular layer of the DG (lower panels). Scale bars: upper panels:400 µm; lower panels: 100 µm. (TIF) [file pone.0063589.s003.tif]
